# Dietary Intake of Potassium and Associated Dietary Factors among Representative Samples of Japanese General Population: NIPPON DATA 80/90

**DOI:** 10.2188/jea.JE20090226

**Published:** 2010-05-05

**Authors:** Tanvir Chowdhury Turin, Nagako Okuda, Katsuyuki Miura, Yasuyuki Nakamura, Nahid Rumana, Hirotsugu Ueshima

**Affiliations:** 1 Department of Health Science, Shiga University of Medical Science, Ohtsu, Japan; 2 Cardiovascular Epidemiology, Kyoto Women’s University, Kyoto, Japan

**Keywords:** nutrition, diet, potassium, mean intake, daily intake, density intake, population

## Abstract

**Objective:**

The purpose of this study was to investigate the dietary potassium intake and associated other dietary factors among a representative sample cohort of Japanese population.

**Methods:**

We obtained data from NIPPON DATA80 and 90 that were conducted with the National Nutrition Surveys in 1980 and in 1990. Then we estimated nutrient and food intakes of individuals in the National Nutrition Survey of 1980 and that of 1990, which were adjusted on the basis of data of the National Nutrition Survey of 1995. We analyzed data for 10 422 participants (4585 men and 5837 women) in NIPPON DATA80 and 8342 participants (3488 men and 4854 women) in NIPPON DATA90 having dietary potassium intake information.

**Results:**

In NIPPON DATA80 and 90 it was observed that there was a significant relationship between the dietary potassium intake and age for both men and women. Higher potassium intake was associated with higher age, intake of protein, iron, calcium, sodium, vitamins, and fiber. Regarding food groups, lower amount of dietary cereals, rice, flour, fats and oils were associated with higher dietary potassium for both men and women. On the other hand, higher intake of nuts, potatoes, soy beans, fruits, vegetables, mushrooms, sea algae, fish and shellfish were associated with higher dietary potassium.

**Conclusions:**

We obtained the mean dietary potassium intake and its association with other dietary nutrient intake in Japanese adults as the baseline data in NIPPON DATA80 and in NIPPON DATA90.

## INTRODUCTION

Adequate dietary potassium intake exerts a blood pressure lowering effect and is associated with a decreased risk of cardiovascular disease.^1^^,^^2^ The beneficial effects of potassium intake also include decreased risk bone demineralization and kidney stones.^3^ Most guidelines on the prevention of chronic diseases and the management of hypertension emphasize the necessity to increase the consumption of potassium-rich foods.^4^^–^^6^ However, little information is available on the dietary potassium intake pattern and its different correlates among Japanese general population. Accumulation of reliable data obtained from cohort studies in Japan, with the highest longevity of the world, using representative population groups is needed to establish strategies in Japan for health promotion and prevention of lifestyle-related diseases that take into account the differences between dietary habits and prevalence of diseases in Japan and other countries.

The National Nutritional Survey (NNS) is household-based food consumption survey with the purposes of obtaining information on the nutritional health, actual food consumption and food requirements in Japan.^7^ Two cohort studies based on the National Survey on Circulatory Disorders in 1980 and 1990,^8^ using the majority of the participants for NNS, have been named as the National Integrated Project for Prospective Observation of Non-communicable Disease and Its Trends in the Aged (NIPPON DATA80 and NIPPON DATA90). Data linkage was performed between NNS and NIPPON DATA with the objective to investigate fundamental data in relation between the dietary nutritional intake and the health.

The purpose of this study was to investigate the dietary potassium intake and associated dietary factors utilizing the aforementioned pooled data which is a representative sample cohort of the Japanese general population.

## PARTICIPANTS AND METHODS

### National nutritional survey

Food intake survey by weighed food records in three consecutive representative days were conducted by specially trained dietary interviewers. These surveys were performed for all household members from randomly selected 300 survey districts throughout Japan. Dietary interviewers visited participants’ houses at least once during the survey. Weekends and holydays were avoided. In the NNS before 1994, nutrient and food intakes per capita were calculated by dividing the amount of food intake by the number of household members. However, since 1995, nutrient and food intakes of individual household members have been calculated by proportional division method for one day, in which the amount of food intake is proportionally divided by the consumption rate of each household member. Average intakes by gender and age class could be calculated by this method. The average intakes in NNS of 1995 were calculated by a combination method of household-based food weighing record and an approximation of proportions by which family members shared each dish or food in the household. In this study, we estimated nutrient and food intakes of individuals in the NNS of 1980 (NNS-80) and that of 1990 (NNS-90), which were adjusted on the basis of data of the NNS of 1995. We estimated nutrient intakes of each household member by dividing household intake data of NNS-80 and -90 proportionally using average intakes by sex and age groups calculated for NNS of 1995.^9^ For each person, means of the estimated individual nutrients from the three days records were used in the analyses. Detailed nutrient that were not included in the NNS were complemented via imputation method. Details of the nutrient intake calculation were descried elsewhere.^10^

### NIPPON DATA80 and 90

Participants for the NIPPON DATA80 and NIPPON DATA90 studies were the residents aged 30 years or older in 300 census tracts, which were selected using the stratified random sampling method, based on the national census of 1975, throughout Japan. Details of these studies have been described elsewhere.^11^^–^^15^ The participants for NIPPON DATA80 and 90 were those persons surveyed for the 3rd and 4th National Survey on Circulatory Disorders, conducted in 1980 and 1990, respectively. During the baseline survey, there were 10 546 participants for NIPPON DATA80 and 8383 participants for NIPPON DATA90. The survey consisted of history taking, physical examinations, blood test, and a self-administered questionnaire on lifestyle, including dietary habit using the food-frequency method. Trained staff at local health centers in the respective districts performed the examinations in community centers.

### Analyses

After excluding the participants who had missing data or total energy intake of less than 500 kcal or more than 5000 kcal, a remaining total of 10 422 participants (men: 4585 & women: 5837) of NIPPON DATA80 and 8342 participants (men: 3488 & women: 4854) of NIPPON DATA90 with dietary potassium intake information were included in the present study. Data were analyzed in men and women separately. Potassium intake was calculated as crude intake (mg per day) and density intake (mg per 1000 kcal). Dietary potassium intake was classified into quintiles and physical, life-style, and dietary parameters were examined across the quintiles. Data are presented as means and standard deviations. Chi-squared tests were used for the categorical variables. To detect differences in continuous variables in groups, analysis of variance (ANOVA) was used. The “contrast” option for analysis of variance was used to detect deviation from linearity in the association between continuous variables and the five potassium intake groups, and trend P was obtained. All statistical analyses were performed using SAS® version 9.1 (SAS Institute, Cary, NC.).

## RESULTS

For the participants of NIPPON DATA80 the mean dietary potassium intake for men and women was 3042.4 (SD 792.3) mg/day and 2768.5 (SD 736.2) mg/day, respectively. The mean dietary potassium density intake was 1274.4 (SD 239.8) mg/1000 kcal for men and 1442.8 (SD 278.3) mg/1000 kcal for women. From the data obtained from NIPPON DATA90, the estimated mean dietary potassium intake for men and women was 3033.5 (SD 804.3) mg/day and 2751.8 (SD 751.4) mg/day, respectively. The mean dietary potassium density intake was 1316.5 (SD 263.4) mg/1000 kcal for men and 1486.0 (SD 302.8) mg/1000 kcal for women.

NIPPON DATA80 participant characteristics and nutrient intakes according to quintiles of dietary potassium intake are shown in Table 1 for men and in Table 2 for women. Higher potassium intake was associated with higher age and intakes of protein, iron, calcium, sodium, and vitamins for both men and women. On the other hand, BMI and total dietary fat did not show any association with the dietary potassium.

**Table 1.** Participant characteristics and nutrient intake according to quintiles of dietary potassium intake for men: NIPPON DATA80

	Potassium intake quintiles (mg/1000 kcal)											
	
Range	548–1079		1080–1195		1196–1310		1311–1452		1453–2433		*P* diff	*P* trend
	
	Mean	*SD*	Mean	*SD*	Mean	*SD*	Mean	*SD*	Mean	*SD*		
*n*	920		915		913		921		916			
Age (year)	45.4	*12.1*	46.8	*12.2*	49.0	*13.0*	52.4	*13.3*	56.1	*13.4*	<0.001	<0.001
BMI (kg/m^2^)	22.4	*2.8*	22.4	*2.9*	22.5	*2.9*	22.5	*2.9*	22.7	*2.9*	0.215	0.593
Current smoking (%)	69.2		66.2		60.5		61.7		57.4		<0.001	
Current drinking (%)	78.9		75.1		75.5		72.5		69.2		<0.001	
Total energy (kcal)	2474.6	*556.7*	2458.7	*488.7*	2416.5	*451.1*	2374.1	*462.9*	2282.3	*521.2*	<0.001	<0.001
Carbohydrate (%kcal)	60.3	*6.4*	59.8	*6.1*	59.5	*6.3*	59.5	*6.2*	59.6	*7.2*	0.021	0.003
Protein (%kcal)	13.6	*1.5*	14.5	*1.6*	15.0	*1.7*	15.6	*1.9*	16.7	*2.3*	<0.001	<0.001
Total fat (%kcal)	20.1	*5.7*	20.2	*5.2*	20.1	*5.0*	20.0	*4.8*	19.5	*5.2*	0.047	0.740
Animal protein (%kcal)	7.5	*1.9*	8.2	*2.1*	8.7	*2.2*	9.0	*2.4*	9.8	*3.1*	<0.001	<0.001
Vegetable protein (%kcal)	6.9	*0.9*	7.1	*0.9*	7.2	*0.9*	7.4	*0.9*	7.8	*1.1*	0.027	0.028
SFA (%kcal)	5.5	*1.5*	5.8	*1.5*	5.7	*1.4*	5.7	*1.4*	5.6	*1.5*	0.007	0.124
MUFA (%kcal)	7.5	*2.1*	7.6	*1.9*	7.5	*1.9*	7.4	*1.8*	7.2	*2.0*	0.002	0.339
PUFA (%kcal)	5.2	*1.6*	5.3	*1.3*	5.3	*1.3*	5.3	*1.3*	5.3	*1.4*	0.870	0.540
Iron (mg/1000 kcal)	5.2	*0.7*	5.8	*0.7*	6.2	*0.8*	6.7	*0.9*	7.5	*1.2*	<0.001	<0.001
Calcium (mg/1000 kcal)	172.7	*40.0*	204.6	*38.2*	225.1	*37.0*	249.7	*42.4*	294.8	*59.2*	<0.001	<0.001
Sodium (mg/1000 kcal)	2019.5	*624.1*	2278.7	*635.1*	2447.0	*674.9*	2672.1	*728.7*	3035.0	*1023.1*	<0.001	<0.001
Vitamin A (IU/1000 kcal)	508.1	*191.1*	637.7	*211.6*	714.2	*236.3*	789.3	*277.9*	983.8	*399.8*	<0.001	<0.001
Vitamin B1 (mg/1000 kcal)	0.4	*0.1*	0.5	*0.2*	0.5	*0.2*	0.5	*0.2*	0.5	*0.2*	<0.001	<0.001
Vitamin B2 (mg/1000 kcal)	0.4	*0.1*	0.5	*0.1*	0.5	*0.1*	0.6	*0.1*	0.6	*0.2*	<0.001	<0.001
Vitamin C (mg/1000 kcal)	29.9	*9.7*	39.1	*10.0*	44.6	*11.7*	52.1	*13.7*	65.9	*20.2*	<0.001	<0.001

*P* diff values obtained by ANOVA or Chi square statistics. *P* trend obtained by contrast statement of analysis of variance. BMI, body mass index; SFA, saturated fatty acid; MUFA, mono unsaturated fatty acid; PUFA, poly unsaturated fatty acid.

**Table 2.** Participant characteristics and nutrient intake according to quintiles of dietary potassium intake for women: NIPPON DATA80

	Potassium intake quintiles (mg/1000 kcal)											
	
Range	481–1211		1212–1348		1349–1484		1485–1648		1649–2847		*P* diff	*P* trend
	
	Mean	*SD*	Mean	*SD*	Mean	*SD*	Mean	*SD*	Mean	*SD*		
*n*	1168		1170		1165		1168		1166			
Age (year)	44.3	*12.3*	46.3	*13.0*	50.0	*13.2*	53.0	*12.9*	56.7	*12.1*	<0.001	<0.001
BMI (kg/m^2^)	22.7	*3.4*	22.7	*3.3*	22.7	*3.4*	22.9	*3.3*	23.2	*3.4*	0.001	0.074
Current smoking (%)	11.8		11.0		8.6		8.8		8.8		0.019	
Current drinking (%)	24.7		22.6		18.6		17.4		18.4		<0.001	
Total energy (kcal)	1987.3	*428.7*	1956.5	*373.5*	1944.5	*371.2*	1910.8	*389.5*	1848.20	*431.5*	<0.001	<0.001
Carbohydrate (%kcal)	62.5	*6.8*	61.7	*6.6*	62.1	*6.6*	62.2	*6.7*	61.8	*7.3*	0.033	0.555
Protein (%kcal)	14.0	*1.5*	14.8	*1.6*	15.4	*1.7*	15.9	*1.9*	17.2	*2.3*	<0.001	<0.001
Total fat (%kcal)	21.9	*6.2*	22.3	*5.8*	21.7	*5.5*	21.5	*5.5*	21.2	*5.8*	<0.001	0.022
Animal protein (%kcal)	7.7	*1.9*	8.4	*2.0*	8.8	*2.2*	9.2	*2.4*	10.1	*3.0*	<0.001	<0.001
Vegetable protein (%kcal)	7.1	*0.9*	7.2	*0.9*	7.4	*0.9*	7.6	*0.9*	8.0	*1.1*	<0.001	0.007
SFA (%kcal)	6.1	*1.7*	6.4	*1.6*	6.2	*1.6*	6.1	*1.6*	6.1	*1.7*	<0.001	0.392
MUFA (%kcal)	8.2	*2.3*	8.3	*2.2*	8.1	*2.0*	8.0	*2.1*	7.9	*2.2*	<0.001	0.007
PUFA (%kcal)	5.7	*1.6*	5.8	*1.5*	5.7	*1.4*	5.7	*1.5*	5.8	*1.6*	0.444	0.409
Iron (mg/1000 kcal)	5.9	*0.8*	6.5	*0.9*	6.9	*0.9*	7.4	*1.0*	8.4	*1.3*	<0.001	<0.001
Calcium (mg/1000 kcal)	209.4	*40.7*	248.1	*42.5*	271.9	*46.4*	297.4	*50.8*	352.8	*70.6*	<0.001	<0.001
Sodium (mg/1000 kcal)	2171.7	*618.8*	2449.6	*696.9*	2630.9	*709.4*	2802.1	*745.8*	3269.3	*1055.6*	<0.001	<0.001
Vitamin A (IU/1000 kcal)	606.0	*229.8*	749.7	*251.0*	828.8	*293.2*	931.9	*322.2*	1168.3	*491.0*	<0.001	<0.001
Vitamin B1 (mg/1000 kcal)	0.5	*0.2*	0.6	*0.2*	0.6	*0.2*	0.6	*0.2*	0.7	*0.2*	<0.001	<0.001
Vitamin B2 (mg/1000 kcal)	0.3	*0.1*	0.3	*0.1*	0.4	*0.1*	0.4	*0.1*	0.5	*0.1*	<0.001	<0.001
Vitamin C (mg/1000 kcal)	39.8	*12.5*	50.9	*12.6*	59.1	*15.2*	68.5	*17.1*	88.2	*26.8*	<0.001	<0.001

*P* diff values obtained by ANOVA or Chi square statistics. *P* trend obtained by contrast statement of analysis of variance. BMI, body mass index; SFA, saturated fatty acid; MUFA, mono unsaturated fatty acid; PUFA, poly unsaturated fatty acid.

Tables 3 and 4 shows the food group intakes according to quintiles of dietary potassium intake for men and women of NIPPON DATA80, respectively. Regarding food groups, lower intakes of dietary cereals, rice, flour, fats and oils were associated with higher dietary potassium for both men and women. On the other hand, higher intake of nuts, potatoes, soy beans, fruits, vegetables, mushrooms, sea algae, fish and shellfish were associated with higher dietary potassium.

**Table 3.** Nutrient intakes of different food group according to quintiles of dietary potassium intake for men: NIPPON DATA80

	Potassium intake quintiles (mg/1000 kcal)											
	
Range	548–1079		1080–1195		1196–1310		1311–1452		1453–2433		*P* diff	*P* trend
	
	Mean	*SD*	Mean	*SD*	Mean	*SD*	Mean	*SD*	Mean	*SD*		
*n*	920		915		913		921		916			
Cereals (g/1000 kcal)	178.7	*30.5*	166.4	*27.0*	160.0	*26.3*	153.2	*26.2*	142.6	*30.2*	<0.001	<0.001
Rice (g/1000 kcal)	139.3	*35.8*	129.2	*32.6*	126.1	*33.5*	123.0	*31.9*	114.2	*34.4*	<0.001	<0.001
Flour product (g/1000 kcal)	42.1	*28.4*	39.8	*24.3*	36.6	*24.3*	33.2	*22.9*	30.3	*23.6*	<0.001	<0.001
Nuts (g/1000 kcal)	0.3	*1.0*	0.6	*1.8*	0.6	*2.2*	0.6	*1.5*	0.7	*1.7*	0.009	<0.001
Potatoes (g/1000 kcal)	16.2	*12.7*	23.2	*13.8*	26.6	*14.9*	31.1	*17.6*	41.3	*25.0*	<0.001	<0.001
Sugar & sweetener (g/1000 kcal)	5.3	*4.0*	5.8	*4.2*	5.7	*4.1*	5.9	*4.0*	5.9	*4.1*	0.007	0.007
Sweet & snacks (g/1000 kcal)	5.8	*6.7*	6.0	*6.1*	6.3	*7.0*	6.7	*7.5*	6.9	*8.4*	0.004	0.005
Fats & Oils (g/1000 kcal)	7.9	*5.1*	7.6	*4.4*	7.1	*4.1*	6.9	*3.9*	6.0	*4.0*	<0.001	<0.001
Soy beans and product (g/1000 kcal)	26.7	*19.3*	31.4	*17.6*	34.1	*19.8*	38.2	*20.9*	43.2	*23.8*	<0.001	<0.001
Fruit (g/1000 kcal)	34.0	*25.0*	48.8	*27.9*	56.1	*31.8*	67.5	*35.9*	89.0	*50.2*	<0.001	<0.001
Green & yellow vegetable (g/1000 kcal)	14.5	*9.9*	19.5	*11.4*	23.1	*12.7*	26.5	*15.4*	35.5	*22.2*	<0.001	<0.001
Other vegetable (g/1000 kcal)	69.7	*23.8*	84.7	*27.2*	92.3	*28.8*	108.0	*34.1*	128.7	*48.9*	<0.001	<0.001
Mushrooms (g/1000 kcal)	3.2	*4.3*	3.4	*4.5*	4.1	*5.3*	4.6	*5.7*	5.5	*6.9*	<0.001	<0.001
Sea algae (g/1000 kcal)	1.5	*1.5*	1.9	*2.1*	2.4	*2.5*	3.1	*3.5*	4.6	*5.4*	<0.001	<0.001
Condiment & beverage (g/1000 kcal)	84.7	*88.2*	75.6	*60.5*	86.5	*79.0*	81.9	*68.9*	82.7	*76.7*	0.025	0.835
Fish & shellfish (g/1000 kcal)	42.2	*20.1*	46.2	*21.4*	51.8	*22.6*	56.3	*23.8*	65.0	*31.6*	<0.001	<0.001
Meat (g/1000 kcal)	30.4	*15.8*	30.6	*15.7*	30.2	*16.6*	28.5	*15.7*	27.7	*18.3*	<0.001	0.014
Egg (g/1000 kcal)	16.1	*9.3*	17.5	*9.2*	17.1	*9.0*	17.4	*9.3*	17.8	*11.0*	0.002	0.017
Milk & dairy (g/1000 kcal)	21.7	*19.8*	27.4	*21.6*	30.7	*21.5*	33.4	*25.8*	38.5	*31.5*	<0.001	<0.001
Other food (g/1000 kcal)	2.7	*5.8*	3.1	*6.9*	2.7	*5.5*	2.8	*6.0*	2.7	*9.7*	0.709	0.952

*P* diff values obtained by ANOVA statistics. *P* trend obtained by contrast statement of analysis of variance.

**Table 4.** Nutrient intakes of different food group according to quintiles of dietary potassium intake for women: NIPPON DATA80

	Potassium intake quintiles (mg/1000 kcal)											
	
Range	481–1211		1212–1348		1349–1484		1485–1648		1649–2847		*P* diff	*P* trend
	
	Mean	*SD*	Mean	*SD*	Mean	*SD*	Mean	*SD*	Mean	*SD*		
*n*	1168		1170		1165		1168		1166			
Cereals (g/1000 kcal)	171.7	*29.1*	159.8	*26.6*	154.4	*26.6*	149.0	*26.1*	137.5	*29.2*	<0.001	<0.001
Rice (g/1000 kcal)	117.3	*32.9*	109.3	*32.3*	110.1	*32.0*	107.8	*32.2*	101.2	*33.0*	<0.001	<0.001
Flour product (g/1000 kcal)	51.8	*30.6*	49.0	*29.3*	42.8	*27.8*	40.0	*26.6*	35.4	*27.3*	<0.001	<0.001
Nuts (g/1000 kcal)	0.7	*3.0*	0.7	*1.9*	0.9	*2.7*	0.9	*2.3*	1.1	*2.7*	<0.001	0.051
Potatoes (g/1000 kcal)	20.5	*14.7*	27.7	*16.4*	31.7	*18.2*	37.6	*21.6*	50.1	*31.3*	<0.001	<0.001
Sugar & sweetener (g/1000 kcal)	6.9	*5.2*	6.9	*4.8*	6.8	*4.7*	6.7	*4.6*	6.7	*4.7*	0.850	0.443
Sweet & snacks (g/1000 kcal)	13.9	*14.4*	13.8	*13.3*	13.3	*13.2*	13.8	*13.8*	11.8	*13.5*	<0.001	0.617
Fats & Oils (g/1000 kcal)	8.8	*5.3*	8.7	*5.0*	7.9	*4.3*	7.6	*4.4*	6.9	*4.5*	<0.001	<0.001
Soy beans and product (g/1000 kcal)	28.8	*19.2*	33.1	*19.0*	37.3	*21.5*	40.7	*22.5*	46.9	*25.8*	<0.001	<0.001
Fruit (g/1000 kcal)	60.2	*38.2*	78.3	*41.9*	94.1	*48.6*	109.6	*58.1*	138.4	*75.4*	<0.001	<0.001
Green & yellow vegetable (g/1000 kcal)	18.4	*12.5*	25.1	*14.2*	29.0	*16.7*	34.6	*19.8*	46.3	*28.7*	<0.001	<0.001
Other vegetable (g/1000 kcal)	78.0	*27.0*	95.5	*29.8*	106.1	*32.8*	120.8	*39.5*	147.3	*53.4*	<0.001	<0.001
Mushrooms (g/1000 kcal)	3.5	*4.8*	3.8	*4.9*	4.5	*5.8*	5.4	*6.3*	6.2	*7.8*	<0.001	<0.001
Sea algae (g/1000 kcal)	1.6	*1.8*	2.2	*2.3*	2.9	*3.1*	3.7	*4.3*	5.5	*6.6*	<0.001	<0.001
Condiment & beverage (g/1000 kcal)	34.5	*36.7*	35.3	*44.7*	35.0	*34.0*	34.7	*32.6*	36.3	*51.0*	0.843	0.964
Fish & shellfish (g/1000 kcal)	40.1	*19.5*	44.7	*20.0*	50.0	*22.4*	53.8	*23.2*	63.3	*29.7*	<0.001	<0.001
Meat (g/1000 kcal)	28.3	*15.3*	28.8	*14.5*	27.8	*15.2*	27.7	*16.2*	27.0	*18.3*	0.092	0.232
Egg (g/1000 kcal)	18.3	*11.1*	18.3	*9.4*	18.3	*9.8*	18.5	*10.0*	18.5	*11.1*	0.927	0.535
Milk & dairy (g/1000 kcal)	35.6	*27.1*	45.6	*31.3*	48.4	*34.0*	50.2	*38.1*	57.4	*47.0*	<0.001	<0.001
Other food (g/1000 kcal)	3.4	*7.2*	3.3	*6.5*	3.1	*6.7*	3.0	*6.3*	3.0	*8.2*	0.660	0.222

*P* diff values obtained by ANOVA statistics. *P* trend obtained by contrast statement of analysis of variance.

Tables 5 and 6 shows participant characteristics and nutrient intakes according to the quintiles of dietary potassium intake for men and women from NIPPON DATA90, respectively. Higher potassium intake was associated with higher age and intake of protein intake, fiber, iron, calcium, sodium, phosphorus, magnesium, and vitamins for both men and women. Intake of fats did not show any association with the dietary potassium. Table 7 and Table 8 show the food groups according to quintiles of dietary potassium intake for men and women of NIPPON DATA90 cohort, respectively. Regarding food groups, lower amount of dietary cereals, rice, flour, fats and oils were associated with higher dietary potassium for both men and women. On the other hand, higher intake of nuts, potatoes, soy beans, fruits, vegetables, sea algae, milk and dairy, and fish and shellfish were associated with higher dietary potassium.

**Table 5.** Participant characteristics and nutrient intake according to quintiles of dietary potassium intake for men: NIPPON DATA90

	Potassium intake quintiles (mg/1000 kcal)											
	
Range	533.6–1090.7		1090.8–1229.8		1229.9–1356.1		1356.2–1518.3		1518.4–2999.7		*P* diff	*P* trend
	
	Mean	*SD*	Mean	*SD*	Mean	*SD*	Mean	*SD*	Mean	*SD*		
*n*	697		698		698		698		697			
Age (year)	47.1	*12.4*	50.7	*12.5*	51.9	*13.7*	55.9	*13.5*	60.8	*12.3*	<0.001	<0.001
BMI (kg/m^2^)	22.8	*3.0*	23.0	*2.9*	22.9	*3.0*	22.9	*3.1*	23.0	*3.1*	0.653	0.610
Current smoking (%)	67.3		59.3		54.7		49.0		42.6		<0.001	
Current drinking (%)	59.0		61.6		55.3		58.2		52.1		0.004	
Total energy (kcal)	2366.4	*483.5*	2364.4	*434.6*	2326.4	*460.4*	2304.3	*448.8*	2218.0	*463.5*	<0.001	0.004
Total carbohydrate (%kcal)	56.3	*5.8*	56.3	*5.4*	56.6	*5.6*	56.9	*5.9*	57.4	*6.1*	0.003	0.049
Dietary fiber (g/1000 kcal)	5.2	*1.0*	6.1	*1.0*	6.9	*1.2*	7.7	*1.4*	9.3	*2.0*	<0.001	<0.001
Protein (%kcal)	14.4	*1.7*	15.1	*1.6*	15.5	*1.6*	16.0	*1.8*	16.8	*2.0*	<0.001	<0.001
Animal protein (%kcal)	8.6	*2.0*	9.2	*2.1*	9.5	*2.0*	9.6	*2.3*	10.0	*2.4*	<0.001	<0.001
Vegetable protein (%kcal)	6.7	*0.8*	6.9	*0.9*	7.1	*0.9*	7.3	*0.9*	7.6	*1.0*	<0.001	<0.001
Total fat (%kcal)	22.6	*4.7*	22.5	*4.4*	22.5	*4.3*	22.2	*4.4*	21.8	*4.6*	0.002	0.068
Animal fat (%kcal)	10.6	*3.1*	10.9	*3.3*	11.0	*3.1*	10.8	*3.3*	11.0	*3.4*	0.069	0.236
Vegetable fat (%kcal)	12.1	*3.7*	11.6	*3.1*	11.6	*3.0*	11.4	*3.0*	10.8	*3.1*	<0.001	<0.001
Dietary cholesterol (mg/1000 kcal)	180.7	*53.5*	180.4	*49.7*	183.6	*50.2*	185.7	*56.7*	188.9	*56.1*	0.014	0.042
SFA (%kcal)	5.8	*1.3*	6.0	*1.3*	6.0	*1.3*	5.9	*1.4*	5.9	*1.5*	0.008	0.027
MUFA (%kcal)	8.1	*1.9*	8.0	*1.7*	8.0	*1.7*	7.8	*1.8*	7.6	*1.8*	<0.001	0.039
PUFA (%kcal)	5.6	*1.4*	5.5	*1.2*	5.6	*1.2*	5.6	*1.3*	5.5	*1.4*	0.539	0.843
Iron (mg/1000 kcal)	4.6	*0.8*	5.1	*0.7*	5.4	*0.8*	5.8	*0.8*	6.6	*1.3*	<0.001	<0.001
Calcium (mg/1000 kcal)	182.6	*46.3*	216.0	*51.8*	231.0	*50.2*	260.7	*58.2*	313.5	*80.4*	<0.001	<0.001
Magnesium (mg/1000 kcal)	112.7	*20.9*	127.3	*24.1*	131.7	*21.3*	143.3	*23.5*	161.8	*31.4*	<0.001	<0.001
Sodium (mg/1000 kcal)	2231.2	*580.8*	2391.0	*573.3*	2531.5	*726.0*	2626.7	*708.4*	2751.3	*747.9*	<0.001	<0.001
Phosphorus (mg/1000 kcal)	520.2	*51.8*	555.1	*53.5*	574.3	*50.1*	600.6	*56.5*	644.4	*66.9*	<0.001	<0.001
Vitamin A (IU/1000 kcal)	880.6	*842.6*	1102.5	*1009.7*	1275.2	*1170.9*	1369.9	*1133.0*	1644.4	*1625.1*	<0.001	<0.001
Vitamin B1 (mg/1000 kcal)	0.5	*0.2*	0.6	*0.2*	0.6	*0.2*	0.6	*0.2*	0.6	*0.2*	<0.001	<0.001
Vitamin B2 (mg/1000 kcal)	0.5	*0.1*	0.6	*0.1*	0.6	*0.1*	0.6	*0.1*	0.7	*0.2*	<0.001	<0.001
Niacin (mg/1000 kcal)	7.7	*1.5*	8.2	*1.5*	8.5	*1.4*	8.8	*1.8*	9.3	*2.0*	<0.001	<0.001
Vitamin C (mg/1000 kcal)	36.2	*22.9*	46.9	*18.9*	53.1	*17.2*	62.3	*18.4*	77.7	*30.7*	<0.001	<0.001
Vitamin D (mg/1000 kcal)	46.0	*52.6*	56.3	*55.7*	54.4	*47.0*	60.6	*54.6*	70.5	*59.0*	<0.001	<0.001
Vitamin E (mg/1000 kcal)	3.9	*0.8*	4.1	*0.8*	4.3	*0.8*	4.5	*0.9*	4.8	*1.0*	<0.001	<0.001

*P* diff values obtained by ANOVA or Chi square statistics. *P* trend obtained by contrast statement of analysis of variance. BMI, body mass index; SFA, saturated fatty acid; MUFA, mono unsaturated fatty acid; PUFA, poly unsaturated fatty acid.

**Table 6.** Participant characteristics and nutrient intake according to quintiles of dietary potassium intake for women: NIPPON DATA90

	Potassium intake quintiles (mg/1000 kcal)											
	
Range	582.8–1232.4		1232.5–1380.5		1380.6–1526.4		1526.5–1713.5		1713.6–3406.6		*P* diff	*P* trend
	
	Mean	*SD*	Mean	*SD*	Mean	*SD*	Mean	*SD*	Mean	*SD*		
*n*	970		971		971		971		971			
Age (year)	46.1	*13.3*	49.4	*14.0*	52.6	*14.2*	56.6	*13.1*	59.4	*11.7*	<0.001	<0.001
BMI (kg/m^2^)	22.5	*3.3*	22.7	*3.5*	22.7	*3.3*	23.1	*3.2*	23.1	*3.3*	<0.001	<0.001
Current smoking (%)	15.4		10.3		7.9		7.8		5.7		<0.001	
Current drinking (%)	8.5		7.7		6.8		5.3		4.1		<0.001	
Total energy (kcal)	1908.9	*379.2*	1865.6	*349.5*	1855.9	*352.3*	1855.0	*377.5*	1808.4	*367.6*	<0.001	0.001
Total carbohydrate (%kcal)	58.0	*6.4*	58.8	*5.9*	58.7	*5.8*	59.3	*5.9*	59.7	*6.5*	<0.001	<0.001
Dietary fiber (g/1000 kcal)	6.2	*1.2*	7.3	*1.2*	8.1	*1.3*	9.2	*1.5*	11.0	*2.3*	<0.001	<0.001
Protein (%kcal)	14.7	*1.7*	15.3	*1.6*	16.0	*1.7*	16.4	*1.8*	17.3	*2.1*	<0.001	<0.001
Animal protein (%kcal)	8.9	*2.0*	9.3	*2.1*	9.8	*2.1*	9.9	*2.3*	10.3	*2.5*	<0.001	<0.001
Vegetable protein (%kcal)	6.9	*0.9*	7.1	*0.8*	7.2	*0.9*	7.5	*0.9*	7.8	*1.0*	<0.001	<0.001
Total fat (%kcal)	25.2	*5.4*	24.6	*5.0*	24.4	*4.8*	24.1	*4.7*	23.7	*5.1*	<0.001	<0.001
Animal fat (%kcal)	11.3	*3.4*	11.4	*3.4*	11.4	*3.3*	11.4	*3.6*	11.5	*3.7*	0.857	0.644
Vegetable fat (%kcal)	14.0	*4.1*	13.2	*3.6*	13.0	*3.3*	12.7	*3.3*	12.2	*3.7*	<0.001	<0.001
Dietary cholesterol (mg/1000 kcal)	196.6	*57.0*	196.2	*58.5*	201.6	*55.1*	201.1	*61.2*	205.0	*63.5*	0.005	0.026
SFA (%kcal)	6.5	*1.5*	6.5	*1.5*	6.5	*1.5*	6.4	*1.5*	6.4	*1.6*	0.259	0.225
MUFA (%kcal)	9.0	*2.1*	8.7	*1.9*	8.7	*1.9*	8.5	*1.9*	8.3	*2.0*	<0.001	<0.001
PUFA (%kcal)	6.2	*1.5*	6.0	*1.3*	6.1	*1.3*	6.0	*1.3*	6.0	*1.5*	0.001	0.010
Iron (mg/1000 kcal)	5.1	*0.9*	5.5	*0.8*	5.9	*0.8*	6.4	*0.9*	7.3	*1.4*	<0.001	<0.001
Calcium (mg/1000 kcal)	219.5	*55.5*	252.4	*55.4*	279.9	*64.6*	308.0	*67.5*	367.0	*92.3*	<0.001	<0.001
Magnesium (mg/1000 kcal)	120.3	*22.9*	133.3	*22.3*	141.1	*23.1*	151.8	*26.1*	173.1	*36.2*	<0.001	<0.001
Sodium (mg/1000 kcal)	2426.7	*597.4*	2601.1	*699.1*	2743.8	*772.9*	2833.3	*766.3*	3013.2	*880.6*	<0.001	<0.001
Phosphorus (mg/1000 kcal)	540.7	*54.2*	574.9	*53.0*	602.5	*53.8*	626.2	*58.5*	672.9	*74.0*	<0.001	<0.001
Vitamin A (IU/1000 kcal)	1081.9	*1183.2*	1231.0	*1035.5*	1375.4	*1090.1*	1555.4	*1219.8*	1929.9	*1955.8*	<0.001	<0.001
Vitamin B1 (mg/1000 kcal)	0.6	*0.2*	0.6	*0.2*	0.6	*0.2*	0.6	*0.2*	0.7	*0.2*	<0.001	<0.001
Vitamin B2 (mg/1000 kcal)	0.6	*0.1*	0.6	*0.1*	0.7	*0.1*	0.7	*0.1*	0.8	*0.2*	<0.001	<0.001
Niacin (mg/1000 kcal)	7.4	*1.4*	7.9	*1.4*	8.3	*1.4*	8.5	*1.6*	9.2	*1.9*	<0.001	<0.001
Vitamin C (mg/1000 kcal)	45.7	*18.7*	60.6	*25.9*	68.4	*20.4*	80.9	*23.9*	100.7	*38.7*	<0.001	<0.001
Vitamin D (mg/1000 kcal)	47.0	*44.4*	53.6	*52.8*	61.3	*53.3*	61.8	*52.2*	74.1	*63.7*	<0.001	<0.001
Vitamin E (mg/1000 kcal)	4.3	*0.9*	4.6	*0.9*	4.8	*0.9*	5.0	*1.1*	5.4	*1.1*	<0.001	<0.001

*P* diff values obtained by ANOVA or Chi square statistics. *P* trend obtained by contrast statement of analysis of variance. BMI, body mass index; SFA, saturated fatty acid; MUFA, mono unsaturated fatty acid; PUFA, poly unsaturated fatty acid.

**Table 7.** Nutrient intakes of different food group according to quintiles of dietary potassium intake for men: NIPPON DATA90

	Potassium intake quintiles (mg/1000 kcal)											
	
Range	533.6–1090.7		1090.8–1229.8		1229.9–1356.1		1356.2–1518.3		1518.4–2999.7		*P* diff	*P* trend
	
	Mean	*SD*	Mean	*SD*	Mean	*SD*	Mean	*SD*	Mean	*SD*		
*n*	697		698		698		698		697			
Cereals (g/1000 kcal)	160.9	*28.5*	149.9	*24.2*	147.2	*27.1*	141.6	*26.2*	134.2	*28.4*	<0.001	<0.001
Rice (g/1000 kcal)	122.5	*32.5*	116.1	*29.2*	111.3	*28.3*	107.9	*29.9*	102.5	*30.8*	<0.001	<0.001
Flour product (g/1000 kcal)	40.1	*26.4*	35.7	*21.7*	36.9	*26.0*	34.3	*24.2*	32.4	*25.0*	<0.001	<0.001
Nuts (g/1000 kcal)	0.3	*1.2*	0.5	*1.5*	0.7	*2.0*	0.7	*1.5*	1.1	*2.4*	<0.001	<0.001
Potatoes (g/1000 kcal)	19.7	*12.1*	24.7	*14.4*	28.0	*14.7*	34.3	*18.2*	41.3	*23.2*	<0.001	<0.001
Sugar & sweetener (g/1000 kcal)	4.8	*3.7*	5.3	*3.5*	5.3	*3.9*	5.3	*3.8*	5.6	*3.9*	0.007	0.025
Sweet & snacks (g/1000 kcal)	5.0	*6.4*	5.8	*7.0*	5.9	*7.8*	6.4	*8.0*	5.7	*8.2*	0.014	0.001
Fats & Oils (g/1000 kcal)	8.5	*4.6*	7.7	*3.7*	7.6	*3.8*	7.2	*3.8*	6.4	*3.6*	<0.001	<0.001
Soy beans and product (g/1000 kcal)	28.6	*18.1*	34.6	*20.9*	36.5	*20.6*	41.0	*20.7*	47.2	*24.1*	<0.001	<0.001
Fruit (g/1000 kcal)	29.7	*26.3*	44.5	*35.0*	52.2	*36.7*	61.7	*39.6*	76.8	*46.8*	<0.001	<0.001
Green & yellow vegetable (g/1000 kcal)	21.6	*12.0*	29.3	*14.4*	36.5	*17.1*	43.1	*19.6*	58.7	*30.3*	<0.001	<0.001
Other vegetable (g/1000 kcal)	61.0	*23.2*	74.1	*24.8*	82.2	*29.2*	93.2	*33.0*	105.0	*40.6*	<0.001	<0.001
Mushrooms (g/1000 kcal)	3.7	*4.5*	4.5	*5.2*	5.4	*5.6*	6.0	*6.4*	7.4	*8.6*	<0.001	<0.001
Sea algae (g/1000 kcal)	2.1	*4.0*	2.4	*2.6*	3.0	*3.7*	3.6	*3.7*	5.4	*5.8*	<0.001	<0.001
Condiment & beverage (g/1000 kcal)	119.5	*111.6*	118.3	*98.0*	98.9	*76.8*	100.4	*89.9*	84.4	*77.1*	<0.001	<0.001
Fish & shellfish (g/1000 kcal)	47.0	*23.3*	52.0	*22.9*	54.5	*23.1*	58.6	*25.1*	62.8	*26.0*	<0.001	<0.001
Meat (g/1000 kcal)	31.7	*16.5*	32.2	*16.1*	31.4	*15.1*	29.4	*16.5*	27.3	*15.8*	<0.001	0.004
Egg (g/1000 kcal)	20.1	*9.8*	19.6	*9.5*	19.4	*9.1*	19.9	*10.2*	19.5	*9.9*	0.595	0.664
Milk & dairy (g/1000 kcal)	25.0	*22.6*	34.0	*25.6*	39.2	*28.3*	42.9	*32.1*	58.5	*47.6*	<0.001	<0.001
Other food (g/1000 kcal)	2.2	*3.5*	1.9	*3.0*	2.2	*3.9*	1.5	*2.6*	1.4	*2.5*	<0.001	0.003

*P* diff values obtained by ANOVA statistics. *P* trend obtained by contrast statement of analysis of variance.

**Table 8.** Nutrient intakes of different food group according to quintiles of dietary potassium intake for women: NIPPON DATA90

	Potassium intake quintiles (mg/1000 kcal)											
	
Range	582.8–1232.4		1232.5–1380.5		1380.6–1526.4		1526.5–1713.5		1713.6–3406.6		*P* diff	*P* trend
	
	Mean	*SD*	Mean	*SD*	Mean	*SD*	Mean	*SD*	Mean	*SD*		
*n*	970		971		971		971		971			
Cereals (g/1000 kcal)	154.4	*28.2*	147.4	*29.1*	141.4	*24.6*	135.7	*25.0*	128.8	*28.5*	<0.001	<0.001
Rice (g/1000 kcal)	103.6	*30.8*	101.4	*30.9*	97.4	*27.9*	96.1	*28.1*	91.3	*29.6*	<0.001	<0.001
Flour product (g/1000 kcal)	48.3	*29.0*	44.2	*26.4*	42.6	*25.9*	37.7	*24.9*	36.4	*27.2*	<0.001	<0.001
Nuts (g/1000 kcal)	0.5	*1.5*	0.7	*2.0*	0.7	*1.9*	1.0	*2.4*	1.3	*2.8*	<0.001	<0.001
Potatoes (g/1000 kcal)	23.8	*14.8*	30.5	*17.4*	34.1	*18.6*	39.7	*22.1*	50.1	*28.1*	<0.001	<0.001
Sugar & sweetener (g/1000 kcal)	5.9	*4.2*	6.3	*4.5*	6.4	*4.4*	6.4	*4.8*	6.5	*4.6*	0.029	0.013
Sweet & snacks (g/1000 kcal)	11.8	*13.6*	12.2	*13.3*	12.2	*13.5*	11.6	*13.6*	10.6	*13.5*	0.057	0.793
Fats & Oils (g/1000 kcal)	9.8	*5.2*	8.8	*4.2*	8.5	*4.0*	7.9	*4.1*	7.3	*4.1*	<0.001	<0.001
Soy beans and product (g/1000 kcal)	31.2	*19.6*	35.1	*20.4*	38.9	*20.2*	43.9	*23.0*	49.1	*26.8*	<0.001	<0.001
Fruit (g/1000 kcal)	48.3	*35.6*	68.3	*45.7*	81.3	*50.2*	98.6	*58.5*	115.8	*66.3*	<0.001	<0.001
Green & yellow vegetable (g/1000 kcal)	28.0	*15.1*	36.6	*18.3*	44.6	*21.2*	54.0	*24.0*	73.4	*36.0*	<0.001	<0.001
Other vegetable (g/1000 kcal)	70.6	*27.2*	84.4	*29.6*	92.5	*32.3*	104.6	*37.6*	118.6	*46.2*	<0.001	<0.001
Mushrooms (g/1000 kcal)	4.4	*6.2*	5.2	*5.9*	6.1	*6.2*	6.7	*7.2*	8.4	*9.6*	<0.001	<0.001
Sea algae (g/1000 kcal)	2.3	*4.2*	3.0	*3.6*	3.6	*4.2*	4.2	*4.3*	6.6	*7.0*	<0.001	<0.001
Condiment & beverage (g/1000 kcal)	49.5	*51.2*	46.8	*36.5*	45.9	*40.0*	41.9	*37.3*	39.6	*34.2*	<0.001	<0.001
Fish & shellfish (g/1000 kcal)	44.3	*19.3*	48.5	*20.8*	54.1	*22.4*	56.2	*24.2*	61.5	*25.2*	<0.001	<0.001
Meat (g/1000 kcal)	31.2	*15.7*	30.4	*15.4*	29.9	*15.3*	28.2	*15.7*	26.7	*16.0*	<0.001	<0.001
Egg (g/1000 kcal)	21.7	*10.5*	21.2	*10.6*	21.1	*10.2*	20.8	*10.8*	20.6	*10.2*	<0.001	<0.001
Milk & dairy (g/1000 kcal)	41.9	*34.1*	52.9	*38.5*	59.1	*41.3*	65.3	*48.4*	78.1	*55.6*	0.219	0.071
Other food (g/1000 kcal)	2.6	*4.0*	2.5	*4.0*	2.1	*3.8*	2.0	*3.5*	1.7	*3.0*	<0.001	<0.001

*P* diff values obtained by ANOVA statistics. *P* trend obtained by contrast statement of analysis of variance.

Table 9 shows the potassium and other nutrients intake by age-group for men and women in cohorts of NIPPON DATA80 and 90, respectively. The dietary potassium intake and major food group intakes were significantly associated with age (all *P*s < 0.001). Similar was also observed for the men and women participants of NIPPON DATA90 (Table 10).

**Table 9.** Potassium and other major nutrient intakes by age group in men and women: NIPPON DATA80

Age	30–39		40–49		50–59		60–69		70–		Trend *P*	*P* diff
Variable	Mean	SD	Mean	SD	Mean	SD	Mean	SD	Mean	SD		

Men (*n*)	1220		1196		1019		679		471			
Potassium (mg/1000 kcal)	1185.0	*202.1*	1239.3	*205.3*	1305.6	*243.6*	1365.4	*254.1*	1397.0	*269.8*	<0.001	<0.001
Potassium (mg/day)	2930.0	*761.3*	3064.9	*742.8*	3237.8	*840.9*	3107.6	*805.8*	2759.8	*738.6*	<0.001	<0.001
Carbohydrate (%kcal)	57.8	*5.8*	58.6	*5.8*	59.7	*6.2*	62.0	*6.5*	64.5	*6.7*	<0.001	<0.001
Protein (%kcal)	14.7	*2.1*	15.0	*2.0*	15.3	*2.2*	15.3	*2.1*	15.1	*2.2*	<0.001	<0.001
Total fat (%kcal)	22.1	*5.1*	20.4	*4.7*	19.4	*5.0*	18.3	*5.0*	17.2	*4.9*	<0.001	<0.001
Total energy (kcal)	2475.2	*486.0*	2476.2	*450.0*	2487.7	*484.2*	2297.8	*541.4*	1981.5	*406.4*	<0.001	<0.001
Women (*n*)	1583		1469		1319		900		566			
Potassium (mg/1000 kcal)	1302.5	*204.3*	1412.2	*249.1*	1521.1	*279.6*	1558.8	*293.2*	1547.6	*314.0*	<0.001	<0.001
Potassium (mg/day)	2546.3	*591.6*	2851.9	*729.9*	3001.9	*772.9*	2830.3	*783.6*	2530.6	*718.7*	<0.001	<0.001
Carbohydrate (%kcal)	59.2	*5.8*	60.9	*6.2*	62.6	*6.5*	65.5	*6.8*	66.4	*7.0*	<0.001	<0.001
Protein (%kcal)	15.1	*1.9*	15.3	*2.1*	15.7	*2.3*	15.7	*2.2*	15.7	*2.3*	<0.001	<0.001
Total fat (%kcal)	23.9	*5.4*	22.8	*5.6*	21.2	*5.4*	18.8	*5.4*	18.5	*5.2*	<0.001	<0.001
Total energy (kcal)	1957.0	*338.5*	2025.5	*400.6*	1984.9	*420.4*	1825.4	*412.8*	1639.2	*341.7*	<0.001	<0.001

Values are in mean and *standard deviation*. Trend *P* obtained by contrast statement of analysis of variance. *P* diff values obtained by ANOVA statistics.

**Table 10.** Potassium and other major nutrient intakes by age group in men and women: NIPPON DATA90

Age	30–39		40–49		50–59		60–69		70–		Trend *P*	*P* diff

Variable	Mean	SD	Mean	SD	Mean	SD	Mean	SD	Mean	SD		
Men (*n*)	660		836		793		708		491			
Potassium (mg/1000 kcal)	1189.7	*196.7*	1245.7	*216.6*	1341.1	*259.6*	1393.9	*218.8*	1455.9	*281.2*	<0.001	<0.001
Potassium (mg/day)	2827.7	*692.9*	2987.8	*714.5*	3269.6	*877.3*	3118.1	*840.9*	2884.7	*800.4*	<0.001	<0.001
Carbohydrate (%kcal)	55.2	*5.1*	54.9	*4.9*	56.2	*5.5*	58.3	*5.8*	60.5	*6.2*	<0.001	<0.001
Protein (%kcal)	15.0	*1.7*	15.6	*1.9*	15.9	*2.0*	15.6	*1.9*	15.7	*2.1*	<0.001	<0.001
Total fat (%kcal)	24.6	*4.2*	23.2	*3.9*	21.9	*4.2*	21.1	*4.6*	20.3	*4.5*	<0.001	<0.001
Total energy (kcal)	2376.7	*436.1*	2405.1	*427.2*	2446.2	*475.3*	2237.8	*414.0*	1984.7	*413.5*	<0.001	<0.001
Women (*n*)	1031		1171		1035		915		702			
Potassium (mg/1000 kcal)	1303.6	*211.5*	1428.6	*258.9*	1566.5	*307.0*	1603.9	*311.2*	1577.5	*318.5*	<0.001	<0.001
Potassium (mg/day)	2446.4	*550.6*	2800.5	*694.9*	3014.2	*825.5*	2896.3	*795.7*	2544.1	*716.1*	<0.001	<0.001
Carbohydrate (%kcal)	55.8	*4.9*	56.9	*5.1*	59.4	*5.8*	61.3	*6.3*	62.7	*6.0*	<0.001	<0.001
Protein (%kcal)	15.3	*1.7*	16.0	*1.9*	16.3	*2.0*	16.1	*2.1*	16.0	*2.1*	<0.001	<0.001
Total fat (%kcal)	27.3	*4.4*	25.8	*4.2*	23.9	*4.7*	22.4	*4.8*	21.2	*4.7*	<0.001	<0.001
Total energy (kcal)	1880.2	*313.9*	1964.7	*350.4*	1926.5	*371.1*	1808.9	*374.7*	1615.5	*325.8*	<0.001	<0.001

Values are in mean and *standard deviation*. Trend *P* obtained by contrast statement of analysis of variance. *P* diff values obtained by ANOVA statistics.

## DISCUSSIONS

This cross-sectional study was initiated to describe the dietary potassium intake and its relation with other dietary factors and clinical characteristics among a representative sample cohort of Japanese population. We found significant relationship between the age and the dietary potassium intake. For both men and women, older aged people had higher potassium. Dietary intake of fruits, vegetables, milk and dairy products were associated with potassium intake where as fat intake did not show any association. On the other hand, lower intake of dietary cereals, rice, flour were associated with higher dietary potassium for both men and women. Higher intake of nuts, potatoes, soy beans, mushrooms, sea algae, fish and shellfish were also associated with higher dietary potassium. Regarding minerals, significant relationship was observed to intakes of iron, calcium, sodium, phosphorus, magnesium, vitamins and dietary potassium intake for both men and women. These dietary patterns had not been highlighted adequately in prior nutrition surveys, and should be confirmed by additional analysis of NIPPON DATA and NNS or other studies. Our findings are potentially useful for the development of hypothesis in future NIPPON DATA studies to examine and interpret interactions in detail between potassium intake and other nutritional factors, food groups, risk factors, comorbidities and mortality from various cardiovascular outcomes.

Potassium, the most abundant intracellular cation, is important for membrane transport, energy metabolism, fluid balance and proper cell functioning. Potassium has a crucial role in membrane polarization and abnormal potassium homeostasis can result in disorders in cardiac, muscle and neurological function.^16^^,^^17^ Available evidences from animal studies, observational epidemiological studies, clinical trials, and meta-analyses of these trials have associated potassium intake with blood pressure.^1^ Higher potassium intake exerts a blood pressure lowering effect. Potassium is present in most food groups, however good sources of potassium include fruits, vegetables, dairy products, whole grains, nuts, seeds and dried beans.^16^

A high potassium intake can be achieved through diet. Because potassium derived from foods is also accompanied by a variety of other nutrients, the preferred strategy to increase potassium intake is to consume foods that are rich in potassium, rather than supplements.^1^ In studies, including the Dietary Approach Against Hypertension (DASH) trial, it was observed that increased fruit and vegetable consumption lowered BP.^18^^–^^20^ DASH has also demonstrated that the addition of low-fat dairy products to a diet high in fruits and vegetables significantly reduced BP.^21^ The effects of potassium on BP depend on the concurrent intake of salt and vice versa. Specifically, an increased intake of potassium has a greater BP-lowering effect in the context of a higher salt intake and lesser BP reduction in the setting of a lower salt intake. Conversely, the BP reduction from a reduced salt intake is greatest when potassium intake is low.^1^ This emphasizes the feasibility of increasing the consumption of potassium-rich foods while reducing that of sodium-rich foods at the individual level. In the generally healthy population with normal kidney function, a high potassium intake from foods poses no risk because excess potassium is readily excreted in the urine. However, in individuals whose urinary potassium excretion is impaired, a higher potassium intake may cause adverse cardiac effects from hyperkalemia.^1^^,^^22^

We observed that the participants with higher intake of dietary potassium tended to be from older age categories. These findings are comparable to the National Nutrition Survey in 2003 findings,^23^ which demonstrated that potassium intake of people aged 50 and over was higher than younger people. The National Nutrition Survey in 2003 was based on the individual nutrition intake data.^23^ Regarding the association between age and dietary potassium intake, similar to our findings, Iso et al^24^ also reported lower intake of potassium among younger age group people using FFQ.

Dietary potassium intake is likely to be associated with the subject level covariates as well as the other components of the dietary patterns of a person. Information about the potassium intake in Japanese population in relation to other nutrient intake and overall dietary habit needs to be further investigated using longitudinal research. While, with the use of the pooled NIPPON DATA and NNS data set it would be possible for us to investigate the interactions in detail between nutritional factors, cardiovascular risk factors and mortality; on the other hand we also should be careful about the mutual correlations between potassium intake, other nutrient and food intake.
